# Developing a PPI inhibitor-based therapy for STXBP1 haploinsufficiency-associated epileptic disorders

**DOI:** 10.3389/fnmol.2014.00006

**Published:** 2014-02-04

**Authors:** Shobbir Hussain

**Affiliations:** ^1^Wellcome Trust – Medical Research Council Cambridge Stem Cell Institute, University of CambridgeCambridge, UK; ^2^Department of Physiology, Development and Neuroscience, University of CambridgeCambridge, UK

**Keywords:** STXBP1, Munc18, syntaxin1a, Ohtahara syndrome, West syndrome, epileptic encephalopathy, infantile spasms, PPI inhibitor

## STXBP1 haploinsufficiency in early onset epileptic encephalopathy

Early onset epileptic encephalopathies that occur in very early childhood are rare but particularly catastrophic forms of epilepsy that are invariably associated with significant neurological morbidity (Nordli, 2012). Mutations in the Syntaxin Binding Protein 1 (STXBP1) gene have been linked with two distinct but related forms of the disorder including early onset epileptic encephalopathy with suppression-bursts (EESB) associated with Ohtahara syndrome, and more recently with infantile spasms (IS) associated with West syndrome (Barcia et al., 2013). Mutations in patients are not inherited but rather found to occur *de novo* in a single copy of the STXBP1 gene. Ohtahara syndrome is the earliest appearing age-related epileptic encephalopathy with seizures first presenting as early as the neonatal period and is diagnosed with a characteristic burst-suppression pattern on EEG (Yamatogi and Ohtahara, 2002). It is an extremely debilitating neurological disorder, involving intractable frequent daily seizures and severe intellectual disability. Patients often do not survive beyond early childhood. West syndrome can present with frequent daily IS-type seizures within the first few weeks but more typically within the first few months of life and is diagnosed by a characteristic hypsarrhythmia pattern on EEG (Wong and Trevethan, 2001). The majority of patients have some degree of developmental delay and go on to have mild to severe intellectual disability. Later in life, symptoms of Ohtahara syndrome patients can sometimes evolve into those usually associated with West syndrome, where the seizure-types become more reminiscent of IS and the burst-suppression pattern on EEG evolves into hypsarrhythmia.

There is currently no cure for Ohtahara syndrome or West syndrome and current therapy, which consists of generic anticonvulsant medication, is largely unsatisfactory due to the refractory nature of the seizures. To date, STXBP1 mutations have been reported in 27 cases of EESB and 7 cases of IS not preceded by EESB/Ohtahara syndrome (Barcia et al., 2013). Whereas most genes associated with epileptic disorders encode ion channels or neurotransmitter receptor subunits, STXBP1 is the first epilepsy-associated gene with a direct role in the neurotransmitter release process (Poduri and Lowenstein, 2011). The presence of STXBP1 protein is necessary for neurotransmitter release in probably all neuron types in the brain (Verhage et al., 2000). However, it may be likely that impaired neurotransmitter release in inhibitory GABAergic interneurons throughout the brain results in uncontrolled synchronous firing of excitatory neurons in regions, resulting in epileptic foci. Indeed, a patient with an STXBP1 mutation was recently reported to have responded well to Vigabatrin (Romaniello et al., 2013), a drug which works specifically by inhibiting the gamma-aminobutyric acid transaminase enzyme responsible for the breakdown of GABA. In this article a potential route toward the development of a targeted anticonvulsant medication for STXBP1-associated epilepsy will be presented. The proposal is based on the refined model of neurotransmitter release suggested by recent findings in the Josep Rizo laboratory (Ma et al., 2013) and also the huge potential held in the field of protein-protein interaction (PPI) inhibitor therapeutic drug design.

## STXBP1 in neurotransmitter release

In neurons, the central molecular machinery involved in mediating rapid exocytosis of neurotransmitter-containing synaptic vesicles are the soluble N-ethylmaleimide-sensitive factor attachment protein receptors (SNAREs) (Rizo and Südhof, 2012). Two of these SNAREs, syntaxin1a and SNAP25 are located within the presynaptic plasma membrane whereas the third, synaptobrevin2 is located within the membrane of the synaptic vesicle. All three proteins contain SNARE domain(s) which can assume alpha-helical conformations when interacting with other SNARE motifs (Fasshauer et al., 1997). The assembly of SNARE motifs from the three proteins into very stable hetero-oligomeric four-helix bundles, known as the SNARE fusion complex, induces fusion of the synaptic vesicle membrane to the presynaptic plasma membrane resulting in neurotransmitter release into the synaptic cleft. However, the detailed molecular mechanisms by which the SNARE fusion complex is assembled and regulated have not been fully elucidated. It is known that syntaxin1a and SNAP25 can form stable SNARE complexes which do not include Synaptobrevin 2, but also consist of four-helix bundles that are constitutively present in the presynaptic plasma membrane; however, these are incapable of participating directly in membrane fusion. These non-productive complexes which result largely from the promiscuity of the syntaxin1a SNARE domain to form stable complexes with other SNARE motifs most likely constitute kinetic traps that hinder SNARE fusion complex assembly (Rizo and Südhof, 2012), and their disassembly is likely important for the liberation of individual monomers that can participate in proper SNARE fusion complex assembly.

The formation of the SNARE fusion complex *in vivo* is also known to require additional factors including STXBP1 (also known as Munc18) and Munc13, and indeed neurotransmitter release is completely abolished in STXBP1-deficient cells (Verhage et al., 2000). However, the role of STXBP1 in neurotransmitter release had been paradoxical since STXBP1 is known to bind tightly to a “closed” conformation of syntaxin1a locking it in this mode inhibitory to SNARE fusion complex assembly (Burkhardt et al., 2008). A recent elegant study more clearly defined the roles of STXBP1 and Munc13 in SNARE fusion complex assembly and has helped refine our model of neurotransmitter release (Ma et al., 2013). The results of Ma et al. showed that STXBP1 is also important for the displacement of SNAP25 from syntaxin1a and the disassembly of the non-productive SNARE complexes. They propose that the resulting binding of STXBP1 to a disassembled syntaxin1a monomer in its closed conformation in fact represents the first active step of fusion complex assembly. Their results also show that Munc13 appears to be critical for transforming syntaxin1a from the “closed” to the “open” confirmation during the actual fusion complex assembly process. Therefore whereas STXBP1 plays a role in disassembling non-productive SNARE complexes and thus capturing individual syntaxin1a monomers in their closed conformation, Munc13 then acts to open syntaxin1a at the right moment so that the correct configuration of SNARE complex, including synaptobrevin2 SNARE motifs, can be formed during the membrane fusion process.

## The potential of drug design targeted for STXBP1 haploinsufficiency

**Figure 1:**
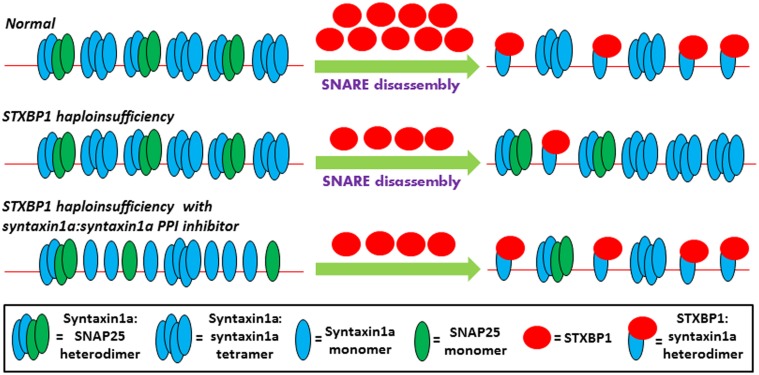
**Theoretical model of the early steps of the neurotransmitter release pathway is depicted in three scenarios.** Under normal conditions, the presynaptic membrane, represented by the thin red line, contains non-productive SNARE complexes which are incapable of directly participating in membrane fusion and include syntaxin1a tetramers (Misura et al., 2001b), and syntaxin1a:SNAP25 heterodimers (Xiao et al., 2001; Misura et al., 2001a). Along with the N-ethylmaleimide-sensitive factor (NSF) and NSF adaptor proteins (Weber et al., 2000), not shown here for the sake of simplicity, STXBP1 is required for the disassembly of the non-productive complexes and the capturing of syntaxin1a monomers in their closed conformation. According to the findings of (Ma et al., 2013), these STXBP1:syntaxin1a heterodimers represent the true starting point of functional SNARE fusion complex assembly, and SNAP25 only becomes re-involved in a later step of the pathway which is not shown here. In STXBP1 haploinsufficiency, the number of functional STXBP1 molecules is reduced, resulting in less efficient disassembly of non-productive SNARE complexes and thus reducing the number of STXBP1:syntaxin1a heterodimers present. The less efficient production of this starting point, which may represent a rate-limiting step of the pathway due to the general stability of SNARE complexes, results in an impairment of eventual neurotransmitter release (not shown here). However, increasing the number of readily-available syntaxin1a monomers, with the use of a syntaxin1a:syntaxin1a PPI inhibitor, may restore the balance of the STXBP1:syntaxin1a starting point formation of fusion complex assembly in STXBP1 haploinsufficiency.

The findings of Ma et al. were most intriguing as it suggested a novel role for STXBP1 in the disassembly of non-productive SNARE complexes and the resulting generation of a productive STXBP1-syntaxin1a sub-complex starting point of SNARE fusion complex assembly. Indeed, due to their stability, the disassembly of the non-productive SNARE complexes may represent a slow rate-limiting step in the pathway toward productive fusion complex assembly. Thus in situations where the number of functional STXBP1 molecules is reduced, such as occurs in STXBP1 haploinsufficieny, progress through the neurotransmitter release pathway is likely to be severely compromised. It may be possible however that increasing the incidence of disassembled syntaxin1a monomers in the presynaptic plasma membrane would counterbalance situations where there is a reduced amount of STXBP1 molecules so that the balance of syntaxin1a-STXBP1 complex formation is restored (Figure 1). One potential mechanism of increasing the number of free syntaxin1a monomers in the presynaptic membrane is to introduce small molecules which can disassemble/inhibit the non-productive SNARE complexes. Such an approach may be especially appealing since if these complexes really are non-functional, then it would follow that the inhibition of these complexes *in vivo* would cause very little side-effects. Another key consideration is the fact that the syntaxin1a-SNAP25 interaction also occurs in the functional SNARE fusion core complex and its structural elements appear largely indistinguishable in nature form its non-productive counterpart (Margittai et al., 2001; Xiao et al., 2001); thus this particular interaction should not be targeted. Instead, the syntaxin1a:syntaxin1a SNARE interaction is unique to the non-productive SNARE complexes and since these complexes are most stable as four-helix bundles, successful disruption of this interaction would most likely lead to destabilization and disassembly of the whole complex. Thus the drug design of a PPI inhibitor which targets the alpha-helical syntaxin1a:syntaxin1a interaction, which occurs specifically within non-productive SNARE complexes, is suggested for the potential use in STXBP1 haploinsufficiency-associated epileptic disorders.

## PPI inhibitors and their ability in targeting alpha helical interactions

Targeting protein-protein interfaces of multiprotein complexes has become a significant focus in drug discovery (Higueruelo et al., 2013). Alpha-helices comprise approximately 40% of all protein secondary structures and they represent fundamental recognition elements in many PPIs. In recent years, significant effort has been directed to develop peptidic and non-peptidic small molecules that are structural or functional mimics of alpha-helices and can be classed into three major categories (Cummings and Hamilton, 2010; Edwards and Wilson, 2011; Azzarito et al., 2013). Type I mimetics are short peptidic oligomers that recapitulate the helical conformation of an interaction interface. Such oligomers can be compared to miniature proteins that occur in nature which are capable of crossing the blood-brain-barrier such as the neurotoxic bee venom peptide apamin (Nicoll et al., 2005). Type II mimetics on the other hand are small non-peptide chemical compounds that bind to a peptide receptor but do not necessarily mimic the original heilx structure and thus serve as functional mimetics. Examples include a family of tetra-substituted imidazoles found to be capable of inhibiting the p53/mDM2 interaction in cancer-associated pathways (Vassilev et al., 2004). Type III mimetics are a class of proteomimetics which match the topography of the original helix by mimicking the spatial orientation of its key recognition residues, rather than recapitulating the helical conformation as observed in type I mimetics. Such an approach has previously been used to inhibit Bcl-2 family interactions (Yin et al., 2005).

## The path ahead

Thus several examples of the successful development of alpha-helical PPI inhibitors have been previously described and the interested reader is referred to previous excellent reviews (Cummings and Hamilton, 2010; Edwards and Wilson, 2011; Azzarito et al., 2013). The molecular structure of the alpha-helical syntaxin1a:syntaxin1a SNARE interface has been characterized using site-directed spin labeling and Electron Paramagnetic Resonance by independent laboratories (Margittai et al., 2001; Xiao et al., 2001; Zhang et al., 2002), and in addition the crystal structure of the syntaxin1a SNARE motif within SNARE complexes has been solved (Sutton et al., 1998). Thus efforts to design a PPI inhibitor for this interaction interface should be possible and may be warranted for potential use in STXBP1 haploinsufficiency-associated disorders. In addition, since transgenic mouse models harboring stxbp1 mutations are also available (Verhage et al., 2000), such a potential PPI inhibitor could readily be tested for its ability to alleviate neurological symptoms *in vivo*. Thus with the combination of clinical genetics, cell biology, and structural biology knowledge at hand, and the potential offered by PPI inhibitors in targeted drug design, we could be on the way to developing a targeted anticonvulsant therapy for the treatment of a particularly devastating class of epileptic disorder.

It is perhaps also noteworthy that in stxbp1-null mice, although neurotransmitter release was completely abolished, neural differentiation and brain assembly occurred normally. But apoptosis in mature neural cells then caused neurodegeneration throughout the brain resulting in death of the null-mice. This may be consistent with the observation that anatomical brain abnormalities can be observed in Ohtahara syndrome patients and less frequently in West syndrome patients (Barcia et al., 2013), and may suggest that apoptosis of mature neural cells also occurs in these patients. However, it is likely that the impeded neurotransmitter release in the developed brain of STXBP1-patients also contributes to their developmental delay and intellectual disability, and this raises the compelling prospect that development of such a syntaxin1a:syntaxin1a PPI inhibitor may have potential use in also limiting these particular phenotypes.
